# Heteromeles Arbutifolia, a Traditional Treatment for Alzheimer’s Disease, Phytochemistry and Safety

**DOI:** 10.3390/medicines3030017

**Published:** 2016-07-07

**Authors:** Xiaogang Wang, Raphael Dubois, Caitlyn Young, Eric J. Lien, James D. Adams

**Affiliations:** 1 Tongji School of Pharmacy, Huazhong University of Science and Technology, Wuhan 430030, Hubei, China; wxg0122@163.com; 2 Department of Pharmacology and Pharmaceutical Sciences, School of Pharmacy, University of Southern California, Los Angeles, CA 90899-9121, USA; rd_820@usc.edu (R.D.); caitlyny@usc.edu (C.Y.); elien@usc.edu (E.J.L.)

**Keywords:** *Heteromeles arbutifolia*, toyon, California holly, Alzheimer’s disease, phytochemistry, safety

## Abstract

**Background:** This study examined the chemistry and safety of *Heteromeles arbutifolia*, also called toyon or California holly, which is a traditional California Indian food and treatment for Alzheimer’s disease. **Methods:** Plant extracts were examined by HPLC/MS, NMR and other techniques to identify compounds. Volunteers were recruited to examine the acute safety of the plant medicine using a standard short-term memory test. **Results:** The plant was found to contain icariside E4, dihydroxyoleanenoic acid, maslinic acid, betulin, trihydroxyoxo-seco-ursdienoic acid, catechin, vicenin-2, farrerol, kaempferide and tetrahydroxyoleanenoic acid. These compounds are anti-inflammatory agents that may protect the blood-brain barrier and prevent inflammatory cell infiltration into the brain. The dried berries were ingested by six volunteers to demonstrate the safety of the medicine. **Conclusion:** The plant medicine was found to contain several compounds that may be of interest in the treatment of Alzheimer’s disease. The plant medicine was found to be safe.

## 1. Introduction

Senile dementia, now commonly called Alzheimer’s disease or vascular dementia, was known among California Indians before Europeans came to California [1]. The condition was commonly treated with a native plant, *Heteromeles arbutifolia*, also called toyon or California holly. The medicine consists of about 5 g of the dried berries which are slowly chewed and swallowed by the patient. The medicine slows down the progression of the disease and helps patients continue to have productive lives. The phytochemistry of the plant has not been adequately addressed, except for two publications that found cyanide in the plant [2,3]. There are reports of cyanide poisoning in insects and goats foraging on the leaves of the plant [3,4]. This indicates that the leaves of the plant may be toxic to some animal species. Most fruits from plants in the Rosaceae family contain cyanide, including apples, apricots, peaches, cherries and plums. The berries of *H. arbutifolia* are eaten fresh and cooked as foods by California Indians and other people [1]. The current study examined the chemistry and safety of the plant medicine.

Several plant-derived compounds are known to be protective in models of Alzhiemer’s disease, including flavonoids, resveratrol, green tea polyphenols, curcumin and ferulic acid [5,6,7,8,9]. Ferulic acid is an antioxidant derived from fruits, vegetables and grains. It prevents beta-amyloid fibril formation and is neuroprotective.

## 2. Experimental Section

Plant material: The leaves of *H. arbutifolia* were collected in May in a canyon near Pasadena, CA, USA, and were used for phytochemical analysis. The fruit of the plant were collected in October in the same location and were made into the plant medicine. The leaves were stored frozen. The fruit were dehydrated with a food dehydrator and stored frozen. 

Preparation of solvent extraction and isolation: The frozen leaves (123 g) were thawed and added to 275 mL of ultrapure water, finely chopped with a blender and extracted with 500 mL of ultrapure acetonitrile to make a preparation for phytochemical analysis. Column chromatography was performed with Silicagel 60 columns (EMD, Darmstadt, Germany) that were developed with the following solvents: ethyl acetate (20%, 30%, then 100%) in hexane. Six fractions were collected and checked by analytical TLC using 20% ethyl acetate in hexane. The plates were sprayed with 10% sulfuric acid and heated to visualize spots. Column purified fractions were further purified by preparative TLC (250 µm thick plates, EMD, Darmstadt, Germany) in 20% or 30% ethyl acetate/hexane. Several bands were scraped from each preparative TLC plate. Some bands were further purified by preparative TLC.

NMR spectra (^1^H and ^13^C NMR) were recorded at room temperature with a Varian Mercury Plus instrument at 400 MHz. Chemical shifts (δ) are reported in ppm relative to TMS. HPLC/MS analysis involved a Thermo Finnigan LCQ DECA (Waltham, MA, USA) with a reverse phase column. The solvent system consisted of 10% MeOH in water that increased at 2% per min to 100% MeOH.

Volunteers were recruited from the community by word of mouth and posters. Each volunteer claimed to be of normal health and no history of dementia. All six volunteers were male and ranged from 21 to 60 years old. Each volunteer was administered the modified mini-mental state test [10], which is routinely used to assess Alzheimer’s disease in patients. They then chewed and swallowed 5 g of the dried berries. After 30 min, they were again administered the mini-mental state test. Each volunteer served as their own control since they were examined before and after ingesting the medicine. There was no blinding of the subjects or investigators, since the purpose was to examine the safety of the medicine.

This work was performed in accordance with the Declaration of Helsinki. Each volunteer signed an informed consent document. All personal information on each volunteer was kept confidential.

## 3. Results 

Several known compounds were identified in the plant extract by HPLC/MS, UV and NMR. Despite reports of cyanide in the leaves of the plant, no cyanogenic compounds were found. The identified compounds were all known compounds. Data for each compound matched published data: icariside E4 [11], 2A,3β-dihdroxyolean 13(18)-en-28-oic acid [12], maslinic acid [13,14,15], betulin [16,17], trihydroxy19-oxo-18,19-seco-urs-11,13(18)-dien-28-oic acid [12], catechin [18], vicenin-2 [18], farrerol [19,20], kaempferide [21], 2A,3α,19α,23-tetrahydroxyolean-12-en-28-oic acid [12], lupeol acetate [22]. The data found in this study are shown below.

### 3.1. Icariside E4

UV/Vis λmax (MeOH) nm (logε): 230, 280.

MS (CI, 70 eV): *m*/*z* (%) = 507 [M + H^+^] (100). 

2A,3β-Dihdroxyolean13(18)-en-28-oic acid.

UV/Vis λmax (MeOH) nm (logε): 235, 280.

MS (CI, 70 eV): *m*/*z* (%) = 495 [M + Na^+^] (60), 391 (100), 383, 149. 

### 3.2. Maslinic Acid

UV/Vis λmax (MeOH) nm (logε): 232, 275.

MS (EI, 70 eV): *m*/*z* (%) = 471 [M^+^] (20), 419 (100). 

### 3.3. Betulin

UV/Vis λmax (MeOH) nm (logε): 235, 265.

^1^H NMR (400 MHz, CDCl3): 0.89, 1.66 (2H, m, CH2), 1.52, 1.57 (2H, m, CH2), 3.0 (1H, m, CH), 1.07 (1H, m, CH), 1.34, 1.47 (2H, m, CH2), 1.36, 1.42 (2H, m CH2), 1.11 (1H, m, CH), 1.21, 1.45 (2H, m, CH2), 1.48, 1.51 (2H, m, CH2), 1.67 (1H, s, CH), 1.07, 1.7 (2H, m, CH2), 1.23, 1.92 (2H, m, CH2), 1.32 (1H, m, CH), 2.2 (1H, m, CH), 1.4, 2.0 (2H, m, CH2), 1.06, 1.86 (2H, m, CH2), 0.96 (3H, s, Me), 0.75 (3H, s, Me), 0.81 (3H, s, Me), 0.91 (3H, s, Me), 0.96 (6H, s, 2Me), 3.2 (2H, m, CH2), 3.66 (3H, s, Me), 4.6, 4.75 (2H, d, CH2, J = 15 Hz).

^13^C NMR (400 MHz, CDCl3): 12.3 (C23), 19.9 (C29), 20.3 (C26), 20.7 (C6), 20.9 (C11), 22.5 (C25), 23.3 (C24), 24.0 (C22), 26.5 (C2), 28.0 (C12), 28.7 (C15), 29.6 (C20), 30.2 (C16), 34.6 (C21), 34.9 (C7), 35.8 (C1), 38.1 (C4), 38.4 (C10), 39.0 (C13), 39.4 (C8), 41.4 (C14), 47.1 (C17), 47.8 (C18), 48.1 (C19), 48.8 (C9), 54.0 (C5), 68.3 (C27), 78.3 (C3), 110.4 (C30), 150.7 (C28).

MS (CI, 70 eV): *m*/*z* (%) = 443 [M+H^+^] (50), 429 [MH-CH_2_^+^] (100).

Trihydroxy19-oxo-18,19-seco-urs-11,13(18)-dien-28oic acid.

UV/Vis λmax (MeOH) nm (logε): 240.

MS (CI, 70 eV): *m*/*z* (%) = 523 [M+Na^+^], 501 [M+H^+^], 439. 

### 3.4. Catechin

UV/Vis λmax (MeOH) nm (logε): 240, 280.

MS (CI, 70 eV): *m*/*z* (%) = 291 [M+H^+^], 161, 147, 139, 123. 

### 3.5. Vicenin-2

UV/Vis λmax (MeOH) nm (logε): 230, 270, 340.

MS (EI, 70 eV): *m*/*z* (%) = 593 [M^+^], 455, 358, 295 

### 3.6. Farrerol

UV/Vis λmax (MeOH) nm (logε): 291, 337.

MS (CI, 70 eV): *m*/*z* (%) = 301 [M+H^+^], 282, 152, 120. 

### 3.7. Kaempferide

UV/Vis λmax (MeOH) nm (logε): 289, 337.

MS (EI, 70 eV): *m*/*z* (%) = 301 [M+H^+^], 258, 210. 

2A,3α,19α,23-Tetrahydroxyolean-12-en-28-oic acid.

UV/Vis λmax (MeOH) nm (logε): 233, 283. 

MS (EI, 70 eV): *m*/*z* (%) = 503 [M^−^], 451. 

### 3.8. Lupeol Acetate

UV/Vis λmax (MeOH) nm (logε): 233, 399.

MS (EI, 70 eV): *m*/*z* (%) = 469 [M+H^+^], 443, 425 [M-Ac], 409, 184.

The safety of the plant medicine was demonstrated in six volunteers. Each volunteer scored 100% on the modified mini-mental state test before and after ingesting 5 g of the berries. No volunteer complained of any adverse effects from eating the berries, which were reported to taste like sweet apples. There were no obvious signs of cyanide poisoning in any individual. The berries of the plant are a traditional food and medicine of California Indians [1].

## 4. Discussion

Several compounds that may be beneficial in the treatment of Alzheimer’s disease were found. A recent paper suggests that Alzheimer’s disease may be caused by damage to the blood-brain barrier which allows macrophages and other inflammatory cells to invade the brain and increase the formation of amyloid plaques and neurofibrillary tangles [23]. 

Icariside compounds, similar to icariside E4, were found in *H. arbutifolia* and are known to protect the blood-brain barrier, prevent the infiltration of inflammatory cells into the brain and prevent neuronal damage [19,24]. An unknown that is either maslinic acid [8,9,10] or pomolic acid [13,25] was found. Maslinic acid is well known to occur in plants in the Rosaceae family, whereas pomolic acid is not. This implies that the compound found in the current study is maslinic acid. Maslinic acid suppresses nuclear factor kappa B which decreases the secretion of tumor necrosis factor α by astrocytes [26]. This is an anti-inflammatory effect that protects the blood-brain barrier since tumor necrosis factor α is involved in stimulating the production of vascular endothelial adhesion factors that increase the adhesion and transmigration of inflammatory cells across the blood-brain barrier [27]. 

Flavonoids seem to be beneficial in the prevention of Alzheimer’s disease [28]. Flavonoids such as catechin were found in the plant and stimulate the non-amyloidogenic cleavage of amyloid precursor protein. Vicenin-2 inhibits the glycation of proteins, an anti-inflammatory effect [29]. Farrerol protects the blood-brain barrier by inhibiting the destruction of endothelial cells through apoptosis [30]. Kaempferide is 4’-methylkaempferol. Kaempferol is neuroprotective in a model of Alzheimer’s disease and reverses amyloid beta–induced neuronal impairment [31]. It remains to be shown if kampferide has similar actions. 

Betulin was a major component in the plant and it prevents sterol regulatory element binding protein activation [32]. It improves insulin resistance and decreases fat build-up in atherosclerosis. By the inhibition of the sterol regulatory element binding protein, it inhibits genes involved in fat accumulation. This may prevent the accumulation of perivascular fat that secretes adipokines such as visfatin. Visfatin may be involved in damaging the blood-brain barrier in Alzheimer’s disease [23]. 

Lupeol acetate was found in the plant and is a triterpene that has anti-inflammatory properties through the modulation of the brain opioid system and tumor necrosis factor α [33]. This is potentially useful in the treatment of Alzheimer’s disease. 

*H. arbutifolia* must not be confused with English holly, *Ilex aquifolium*. Although both plants have red berries and spiny leaves, *I. aquifolium* berries are poison, whereas *H. arbutifolia* berries are not. 

## 5. Conclusions

The traditional medicine *H. arbutifolia* has a number of active compounds that are potentially beneficial in Alzheimer’s disease. This plant medicine may provide new leads for drug therapy in the disease. The phytochemistry of the plant indicates that protection of the blood-brain barrier and prevention of inflammatory cell infiltration into the brain may be important targets in the treatment of Alzheimer’s disease. It may be worthwhile to investigate the use of the plant medicine itself in Alzheimer’s disease.

## References

[1] Garcia C., Adams J. (2012). Healing with Medicinal Plants of the West Cultural and Scientific Basis for Their Use.

[2] Dement W., Mooney H. (1974). Seasonal variation in the production of tannins and cyanogenic glucosides in the chaparral shrub *Heteromeles arbutifolia*. Oecologia.

[3] Dahlman D., Johnson V. (1980). *Heteromeles arbutifolia* (Rosaceae: Pomoideae) found toxic to insects. Entomol. News.

[4] Teqzes J., Puschner B., Melton L. (2003). Cyanide toxicosis in goats after ingestion of California Holly (*Heteromeles arbutifolia*). J. Vet. Diagn. Investig..

[5] Pocernich C., Lange M., Sultana R., Butterfield D. (2011). Nutritional approaches to modulate oxidative stress in Alzheimer’s disease. Curr. Alzheimer Res..

[6] Picone P., Bondi M., Montana G., Bruno A., Pitarresi G., Giammona G., di Carlo M. (2009). Ferulic acid inhibits oxidative stress and cell death induced by Ab oligomers: Improved delivery by solid lipid nanoparticles. Free. Radic. Res..

[7] Ono K., Hirohata M., Yamada M. (2005). Ferulic destabilizes preformed beta-amyloid fibrils in vitro. Biochem. Biophys. Res. Commun..

[8] Sgarbossa A., Giacomazza D., di Carlo M. (2015). Ferulic acid: A hope for Alzheimer’s disease therapy from plants. Nutrients.

[9] Jung J., Yan J., Li H., Sultan M., Yu J., Lee H., Shin K., Song D. (2016). Protective effects of a dimeric derivative of ferulic acid in animal models of Alzheimer’s disease. Eur. J. Pharmacol..

[10] Teng E., Chui H. (1987). The modified mini-mental state (3MS) examination. J. Clin. Psychiatry.

[11] Miyase T. (1989). Ionone and lignan glycosides from *Epimedium diphyllum*. Phytochemistry.

[12] Zeng N., Shen Y., Li L., Jiao W., Gao P., Song S., Chen W., Lin H. (2011). Antiinflammatory triterpenes from the leaves of *Rosa laevigata*. J. Nat. Prod..

[13] Numata A. (1989). Cytotoxic triterpenes from a Chinese medicine, Goreishii. Chem. Pharm. Bull..

[14] Hossain M., Ismail Z. (2013). Isolation and characterization of triterpenes from the leaves of *Orthosiphon stamineus*. Arab. J. Chem..

[15] Chaudhuri P., Singh D. (2013). A new triterpenoid from the rhizomes of *Nelumbo nucifera*. Nat. Prod. Res..

[16] Hayek E., Jordis U., Moche W., Sauter F. (1989). A bicentennial of betulin. Phytochemistry.

[17] Kim D., Chen Z., Nguyen V., Pezzuto J., Qiu S., Lu Z. (1997). A concise semi-synthetic approach to betulinic acid from betulin. Synth. Commun..

[18] Grayer R., Kite G., Abou-Zaid M., Archer L. (2000). The application of atmospheric pressure chemical ionization liquid chromatography-mass spectrometry in the chemotaxonomic study of flavonoids: Characterization of flavonoids from *Ocimum gratissimum* var. *gratissimum*. Phytochem. Anal..

[19] Youssef D., Ramadan M., Khalifa A. (1998). Acetophenones, a chalcone, a chromone and flavonoids from *Pancratium maritimum*. Phytochemistry.

[20] Iwashina T., Kitamjima J., Matsumoto S. (2006). Flavonoids in the species of *Cyrtomium* (Dryopteridaceae) and related genera. Biochem. Syst. Ecol..

[21] Baracco A., Bertin G., Gnocco E., Legorati M., Sedocco S., Catinella S., Favretto D., Traldi P. (1995). A comparison of the combination of fast-atom bombardment with tandem mass spectrometry and of gas chromatography with mass spectrometry in the analysis of a mixture of kaempferol, kaempferide, luteolin and oleuropein. Rapid Commun. Mass Spectrom..

[22] Saratha V., Pillai I., Subramanian S. (2011). Isolation and characterization of lupeol, a triterpenoid from *Calotropis gigantea* latex. Int. J. Pharm. Sci. Rev. Res..

[23] Adams J. (2008). Alzheimer’s disease, ceramide, visfatin and NAD. CNS Neurol. Disord. Drug. Targets.

[24] Yan B., Pan C., Mao X., Yang L., Liu Y., Yan L., Mu H., Wang C., Sun K., Liao F. (2014). Icariside II improves cerebral microcirculatory disturbance and alleviates hippocampal injury in gerbils after ischemia reperfusion. Brain Res..

[25] Lee T., Juang S., Hsu F., Wu S. (2005). Triterpene acids from the leaves of *Planchonella duclitan* (Blanco) Bakhuizan. J. Chin. Chem. Soc..

[26] Huang L., Guan T., Qian Y., Huang M., Tang X., Li Y., Sun H. (2011). Anti-inflammatory effects of maslinic acid, a natural triterpene, in cultured cortical astrocytes via suppression of nuclear factor-kappa B. Eur. J. Pharmacol..

[27] Tak P., Taylor P., Breedveld F., Smeets T., Daha M., Kluin P., Meinders A., Maini R. (1996). Decrease in cellularity and expression of adhesion molecules by anti-tumor necrosis factor α monoclonal antibody treatment in patients with rheumatoid arthritis. Arthritis Rheum..

[28] Mandel S., Youdim M. (2004). Catechin polyphenols: Neurodegeneration and neuroprotection in neurodegenerative diseases. Free Radic. Biol. Med..

[29] Islam N., Ishita I., Jung H., Choi J. (2014). Vicenin-2 isolated from Artemisia capillaris exhibited potent anti-glycation properties. Food Chem. Toxicol..

[30] Li J., Ge R., Tang L., Li Q. (2013). Protective effects of farrerol against hydrogen-peroxide-induced apoptosis in human endothelium-derived EA.hy926 cells. Can. J. Physiol. Pharmacol..

[31] Kim J., Choi S., Cho H., Hwang H., Kim Y., Lim S., Kim C., Kim H., Peterson S., Shin D. (2010). Protective effects of kaempferol (3,4′,5,7-tetrahydroxyflavone) against amyloid beta peptide (Abeta)-induced neurotoxicity in ICR mice. Biosci. Biotechnol. Biochem..

[32] Tang J., Li J., Qi W., Qiu W., Li P., Li B., Song B. (2011). Inhibition of SREBP by a small molecule, betulin, improves hyperlipidemia and insulin resistance and reduces atherosclerotic plaques. Cell Metab..

[33] Lucetti D., Lucetti E., Bandeira M., Veras H., Silva A., Leal L., Lopes A., Alves V., Silva G., Brito G. (2010). Anti-inflammatory effects and possible mechanism of action of lupeol acetate isolated from Himatanthus drasticus (Mart) Plumel. J. Inflamm..

